# Response of soil bacterial communities in wheat rhizosphere to straw mulching and N fertilization

**DOI:** 10.3389/fmicb.2022.982109

**Published:** 2022-12-09

**Authors:** Songhe Chen, Xiaoling Xiang, Hongliang Ma, Petri Penttinen, Ting Zheng, Xiulan Huang, Gaoqiong Fan

**Affiliations:** ^1^Key Laboratory of Crop Eco-physiology and Farming System in Southwest China, Ministry of Agriculture, College of Agronomy, Sichuan Agricultural University, Chengdu, Sichuan, China; ^2^Root Biology Center, College of Resources and Environment, Fujian Agriculture and Forestry University, Fuzhou, China; ^3^Department of Microbiology College of Resources, Sichuan Agricultural University, Chengdu, Sichuan, China

**Keywords:** rhizosphere bacterial community, gene abundance, straw mulching, N fertilization, 16S rRNA

## Abstract

Straw mulching and N fertilization are effective in augmenting crop yields. Since their combined effects on wheat rhizosphere bacterial communities remain largely unknown, our aim was to assess how the bacterial communities respond to these agricultural measures. We studied wheat rhizosphere microbiomes in a split-plot design experiment with maize straw mulching (0 and 8,000 kg straw ha^−1^) as the main-plot treatment and N fertilization (0, 120 and 180 kg N ha^−1^) as the sub-plot treatment. Bacterial communities in the rhizosphere were analyzed using 16S rRNA gene amplicon sequencing and quantitative PCR. Most of the differences in soil physicochemical properties and rhizosphere bacterial communities were detected between the straw mulching (SM) and no straw mulching (NSM) treatments. The contents of soil organic C (SOC), total N (TN), NH_4_^+^-N, available N (AN), available P (AP) and available K (AK) were higher with than without mulching. Straw mulching led to greater abundance, diversity and richness of the rhizosphere bacterial communities. The differences in bacterial community composition were related to differences in soil temperature and SOC, AP and AK contents. Straw mulching altered the soil physiochemical properties, leading to greater bacterial diversity and richness of the rhizosphere bacterial communities, likely mostly due to the increase in SOC content that provided an effective C source for the bacteria. The relative abundance of Proteobacteria was high in all treatments and most of the differentially abundant OTUs were proteobacterial. Multiple OTUs assigned to Acidobacteria, Chloroflexi and Actinobacteria were enriched in the SM treatment. Putative plant growth promoters were enriched both in the SM and NSM treatments. These findings indicate potential strategies for the agricultural management of soil microbiomes.

## Introduction

Wheat (*Triticum aestivum* L.) is a vital part of global food security; approximately 40% of the world’s population get part of their daily nutrients from wheat (Giraldo et al., 2019). Like other plants, wheat hosts a diverse microbial community on its rhizosphere; the rhizosphere microbial communities are closely involved in the cycling of nutrients, plant growth promotion, and resource utilization efficiency of plants (Mendes et al., 2013; Pérez-Montaño et al., 2014). The rhizosphere microorganisms are sensitive to changes in environmental conditions, e.g., to changes brought on by agricultural management (Mendes et al., 2013; Bay et al., 2021). Therefore, it is important to understand how agricultural management measures affect the wheat rhizosphere microbial community and the relationship between the wheat rhizosphere microbial community composition and wheat yield.

Straw mulching is a common agricultural practice that can alleviate soil degradation caused by long-term synthetic fertilizer application and reduce the environmental footprint of food production (Sun et al., 2015; Chai et al., 2021). Straw mulching improved the soil structure and increased organic C content (Blanco-Canqui and Lal, 2007), soil enzyme activity (Zhang et al., 2016; Akhtar et al., 2019a), soil water content (Akhtar et al., 2019b), and other physicochemical properties in topsoil (Dai et al., 2019). However, results on the responses of soil bacteria to straw mulching in the topsoil have been inconsistent (Chen et al., 2017; Liu C. et al., 2020; Qiu et al., 2020; Wang H. H. et al., 2020; Yan et al., 2020), possibly due to different planting systems and regional differences. For example, mulching affected β-diversity but not α-diversity in maize and rice fields in northeast China (Wang H. H. et al., 2020; Yan et al., 2020). In rice-maize rotation systems on three sites in central to eastern China, mulching increased the microbial biomass on every site, but the abundances of gram-negative bacteria, gram-positive bacteria and actinomycetes were higher under mulching on one site only (Chen et al., 2017). In a spring wheat–pea rotation in northwest China, without tillage the α-diversity of bacteria was slightly higher in the no straw mulching treatment, whereas with tillage, the diversity was higher in the mulched treatment (Liu C. et al., 2020). Thus, even though straw mulching has been shown to improve soil quality and crop yield (Dong et al., 2018), its varying effects on the rhizosphere communities call for studies at many crop-region combinations.

N fertilization, one of the important limiting nutrients for plants and microorganisms, is supplied worldwide into terrestrial ecosystems (Liu W. B. et al., 2020). In long-term fertilization experiments, the abundance and diversity of bacteria decreased with increasing N fertilizer levels (Zhou et al., 2015). According to a meta-analysis, N addition decreased microbial diversity and the relative abundances of *Actinobacteria* and *Nitrospirae* (Wang et al., 2018). N enrichment affected the composition and functions of soil microbial communities and reduced microbial diversity (Zhou et al., 2015; Ling et al., 2017); the changes were mainly associated with soil pH and soil organic C and total N contents (Rousk et al., 2010; Kong et al., 2019). While the effect of N fertilization on the nutrient contents and soil bacterial communities has been demonstrated, the responses of rhizosphere bacteria to combined straw mulching and N fertilization has been rarely studied.

We studied soil physicochemical properties and wheat rhizosphere microbiomes using a split-plot design experiment with straw mulching as the main plot treatment and N fertilization as the sub-plot treatment. Bacterial communities in the rhizosphere were analyzed using 16S rRNA gene amplicon sequencing and quantitative PCR. We hypothesized that the N fertilization related abundance and diversity decrease would be alleviated by straw mulching and expected that higher shoot biomass and yield of wheat would be associated with higher abundance and α-diversity of bacteria.

## Materials and methods

### Experimental design and sampling

The study was conducted at the Renshou experimental base (30°04′ N, 104°13′ E) at the College of Agronomy, Sichuan Agricultural University, China. The region belongs to the subtropical monsoon climatic zone in which the winter wheat-summer maize rotation system is one of the major agricultural production systems. The soil is classified as Regosol in the FAO Soil Taxonomy, and the soil texture is loamy clay. At the start of the experiment in 2015, the physical and chemical properties of the topsoil (0–20 cm) were as follows: pH, 7.82; soil organic C content, 9.78 g kg^−1^; total N content, 0.83 g kg^−1^; total phosphorus content, 0.86 g kg^−1^; and total potassium content, 13.96 g kg^−1^.

The experimental design was the same as in our previous study (Chen S. H. et al., 2021). Briefly, the split-plot experimental design consisted of maize straw mulching treatments in the main plot and N fertilization levels in the subplot, with four 30 m^2^ (6 m × 5 m) replicate plots and a total of 24 experimental plots. The experiment was started in 2015. The straw mulching treatments were no straw mulching (NSM) and straw mulching with 8,000 kg straw ha^−1^ (SM). In the SM treatment, the maize residue was chopped and spread on the soil surface after the maize harvest at the end of August each year. The N fertilization levels were 0 (N0), 120 (N1) and 180 kg N ha^−1^ (N2). All the plots were fertilized with P at 75 kg P_2_O_5_ ha^−1^ and K at 75 kg K_2_O ha^−1^. Winter wheat (*Triticum aestivum* L. cv. Chuanmai104) was sown with no-tillage in late October at a basic seedling rate of 2.25 × 10^6^ plant ha^−1^ with 20 cm row spacing and 10 cm hole spacing. Wheat was harvested the following year in May. The field management was based on the local farmers’ management model with no tillage, natural rainfall irrigation, and herbicide treatment once before sowing the wheat.

Rhizosphere soils were collected on 20 March, 2019, at the anthesis stage of winter wheat. Whole plants were excavated using a clean spade and gently shaken to remove the soil not tightly attached to the root (Mapelli et al., 2018; Wang Z. T. et al., 2020). The root systems were separated from the plant and collected in sterile plastic bags. The rhizosphere soil particles were first detached from the roots using the pull and shake method. The tightly attached soil was detached by placing the roots in sterile tubes containing 9 ml of 9 g L^−1^ NaCl, vortexing the tubes for 5 min and centrifuging at 8000 rpm for 10 min, after which the supernatant was discarded and the remaining soil was merged with the rhizosphere soil particles (Mapelli et al., 2018). The rhizosphere soil samples were stored at −80°C for molecular analysis.

### Soil physico-chemical analysis

Air-dried soil was used for physical–chemical analysis. Soil pH was determined in a 1:2.5 soil/water mixture. Soil organic C (SOC) was determined using potassium dichromate oxidation external heating method. Soil total N (TN) and available N (AN) were determined using the alkaline hydrolysis diffusion method and semi-micro Kjeldahl method, respectively (Lu, 1999). Soil available phosphorus (AP) was extracted using sodium bicarbonate and measured using the molybdenum blue method (Lu, 1999). Soil available potassium (AK) was extracted using ammonium acetate and determined with flame photometry (P7 Double Beam UV–Visible Spectrophotometer; MAPADA Inc. Shanghai, China; Lu, 1999). Soil extractable ammonium (NH_4_^+^) and nitrate (NO_3_^−^) were extracted with 2 mol L^−1^ KCl and determined colorimetrically (Liu C. et al., 2020). To estimate the average temperature of soil, the temperature of soil at 10 cm depth was measured from 8 a.m. to 20 p.m. every 2 h with a moisture and water potential meter (em50g, Decagon Devices Inc. Pullman, USA) when collecting soil samples.

### DNA extraction and sequencing

DNA was extracted from 0.5 g fresh soil using a DNeasy Powersoil kit (Qiagen, Manchester, UK) following the manufacturer’s instructions. The concentration and purity of the extracted DNA was assessed using a Nanodrop spectrophotometer (Thermo Fisher Scientific, Waltham, MA, USA) and agarose gel electrophoresis. The DNA samples were stored at −20°C until further processing.

The copy number of the 16S rRNA gene was determined using real-time quantitative PCR (qPCR) on a ABI7500 Fast Real-Time PCR System (Applied Biosystems Inc., USA). 16S rRNA gene fragments were amplified in triplicate using primers 319f (5′-ACTCCTACGGGAGGCAGCAG-3′) and primer 806r (5′-GGACTACHVGGGTWTCTAAT-3′; Walters et al., 2011). Amplification was done in a 20 μl reaction including 10 μl SYBR Green Master Mix (Applied Biosystems, USA), 0.25 μl (10 μM) of each primer, 1 μl (1-10 ng) DNA template or 1 μl sterilized distilled water in the negative control, and 8.5 μl double distilled water (ddH_2_O). Amplification was initiated by denaturation at 95°C for 5 min, followed by 40 cycles of denaturation at 95°C for 15 s, annealing at 60°C for 30s, extension at 72°C for 30 s, and reading the plate at 80°C.

The standards for qPCR were made by cloning a bacterial gene fragment amplified as described above using pmD®18-T Vector (TaKaRa, Dalian, China) according to manufacturer’s instructions Plasmid DNA was extracted using a Plasmid Miniprep Kit (BIOMIGA, Santiago, USA), and the plasmid concentration was measured with a spectrophotometer (Nanodrop 2000, Thermo Scientific, Wilmington, USA). As the sequences of the vector and PCR inserts were known, bacterial gene copy number was calculated directly from the concentration of extracted plasmid DNA. Standard curves were generated using triplicate dilution series of plasmid with a cloned target gene, from 10^3^ to 10^9^ copies of the template. PCR efficiency was 91.3% with *R*^2^ value of 0.999 and slope of −3.55. No amplification was detected in the negative controls.

The V3-V4 region of bacterial 16S rRNA gene was amplified from the soil DNA extracts using the primers 319F and 806R with 7-bp barcodes specific for the samples for multiplex sequencing. The amplification reaction mixture contained 5 μl of Q5 reaction buffer (5×), 5 μl of Q5 High-Fidelity GC buffer (5×), 0.25 μl of Q5 High-Fidelity DNA Polymerase (5 U μl^−1^), 2 μl of dNTP (2.5 mM), 1 μl of each forward and reverse primer (10 μM), 2 μl of DNA template, and 8.75 μl of ddH_2_O. The amplification conditions were initial denaturation for 2 min at 98°C, 25 cycles of denaturation at 98°C for 15 s, annealing at 55°C for 30 s and extension at 72°C for 30 s, and a final extension for 5 min at 72°C. PCR products were purified using Agencourt AMPure XP Beads (Beckman Coulter, Indianapolis, IN, USA), quantified using the PicoGreen dsDNA Assay Kit (Invitrogen, Carlsbad, CA, USA), and pooled in equimolar concentrations. Amplicons were sequenced using paired-end 2 × 300 bp sequencing on Illumina MiSeq at Shanghai Personal Biotechnology Co., Ltd. (Shanghai, China). The sequences have been deposited in the NCBI Sequence Read Archive database under the accession number SRP297976.

### Bioinformatics

Sequences were processed using QIIME2 2019.4^1^ with modifications as described in the official tutorials. The unique sequences were clustered at 98% sequence identity and, after removal of chimeras, the 826,884 high-quality sequences from 24 samples were reclustered into operational taxonomic units (OTUs) at 97% identity. After removing singletons, taxonomy was assigned to OTUs using the classify-sklearn naïve Bayesian taxonomy classifier in feature-classifier plugin (Wang et al., 2007) against the SILVA Release 132 Database (Quast et al., 2013).

### Statistical analysis

Differences in soil properties were tested using two-way ANOVA followed by Fisher’s Least Significant Difference (LSD) test in SPSS 26 (IBM, Armonk, New York, USA). α-diversity indices were visualized using Origin2017 (Origin, OriginLab Inc. MA, USA) and tested using Kruskal Wallis test and Dunn’s test as a *post hoc* test. Taxonomic compositions were visualized using MEGAN (Huson et al., 2011) and GraPhlAn (Asnicar et al., 2015). β-diversity was visualized using principal coordinate analysis (PCoA) in the R package “vegan” in R v.4.1.0 (Oksanen et al., 2019; R Core Team, 2020). Differences in β-diversity were tested using repeated measures permutational multivariate analysis of variance (PERMANOVA) based on Bray–Curtis dissimilarity (Bray and Curtis, 1957; Anderson et al., 2008). Differential abundance analysis at OTU level was done using Linear Discriminant Analysis (LDA) Effect Size (LEfSe; Segata et al., 2011).^2^ OTUs with an LDA score ≥ 2.0 were considered differentially abundant. The relationships between soil properties and bacterial communities were tested based on Bray-Curtis dissimilarities, using distance-based redundancy analysis (RDA) with 9,999 permutations in the vegan R package in R v.4.1.0. The relationships between the soil microbial features and soil properties were tested using Mantel test in the ggcor package in R v.4.0.5.^3^ The correlations among α-and β-diversities and soil characteristics were calculated and visualized using the corrplot package in R.^4^

## Results

### Soil physicochemical analysis

The contents of soil organic C (SOC), total N (TN), NH_4_^+^-N, available N (AN), available P (AP) and available K (AK) were higher in the mulched treatment (SM) than in the non-mulched (NSM) treatment (*p* < 0.05; Table 1). The contents of TN, AN, NH_4_^+^-N, NO_3_^−^-N and AP were higher in the 120 (N1) and 180 kg N ha^−1^ (N2) treatments than in the 0 kg N ha^−1^ (N0) treatment (*p* < 0.05; Table 1). The SOC, TN, AN, NH_4_^+^-N, AP and AK contents were lowest in NSMN0 and highest in SMN2.

**Table 1:** Physicochemical properties of wheat rhizosphere soil in the straw mulching and N fertilization treatments.

Treatments		pH	SOC	TN	AN	NH_4_^+^-N	NO_3_^−^-N	AP	AK
			(g kg^−1^)	(g kg^−1^)	(mg kg^−1^)	(mg kg^−1^)	(mg kg^−1^)	(mg kg^−1^)	(mg kg^−1^)
NSM	N0	8.02^a^	9.12^a^	0.87^b^	57.3^b^	1.84^b^	6.58^c^	7.51^b^	164^b^
	N1	8.06^a^	9.71^a^	1.02^a^	63.3^a^	3.54^a^	12.46^b^	8.41^a^	174^a^
	N2	8.04^a^	9.64^a^	1.03^a^	64.2^a^	3.78^a^	14.54^a^	8.26^a^	171^a^
	Mean	8.04	9.49	0.97	61.6	3.05	11.2	8.06	170
SM	N0	8.01^a^	13.62^ab^	0.94^b^	61.5^c^	2.85^b^	5.58^c^	8.44^b^	198^a^
	N1	7.98^a^	12.98^b^	1.05^a^	64.5^b^	4.36^a^	9.23^b^	9.19^a^	203^a^
	N2	8.06^a^	13.9^a^	1.08^a^	69.3^a^	4.45^a^	10.91^a^	9.44^a^	201^a^
	Mean	8.02	13.5	1.02	65.1	3.89	8.6	9.02	200
	*F*-value and significances									
		pH	SOC	TN	AN	NH_4_^+^-N	NO_3_^−^-N	AP	AK
*M*		0.83	158.23^**^	65.13^**^	341.59^**^	91.90^**^	222.44^**^	23.90^*^	1688.10^**^
*N*		0.77	1.56	128.06^**^	50.32^**^	223.96^**^	280.95^**^	26.72^**^	6.75^*^
*M* × *N*		1.94	2.97	2.6	3.85	1.75	11.42^**^	1.13	1.00

NSM, no straw mulching; SM, straw mulching; N0, no N; N1, 120 kg N ha^−1^; N2, 180 kg N ha^−1^; SOC, Soil organic C; TN, Total N; AN, Available N; NH_4_^+^-N, Ammonium N; NO_3_^−^-N, Nitrate N; AP, Available phosphorus; AK, Available potassium. Values (mean ± SD, n = 4) followed by different superscript letters in a column indicate statistically significant differences between N fertilization levels within mulching treatments (P < 0.05). ** and * indicate statistically significant differences between mulching (M) treatments and N (N) treatments at P < 0.01 and p < 0.05, respectively.

### Abundance and composition of bacterial community

**Figure 1:**
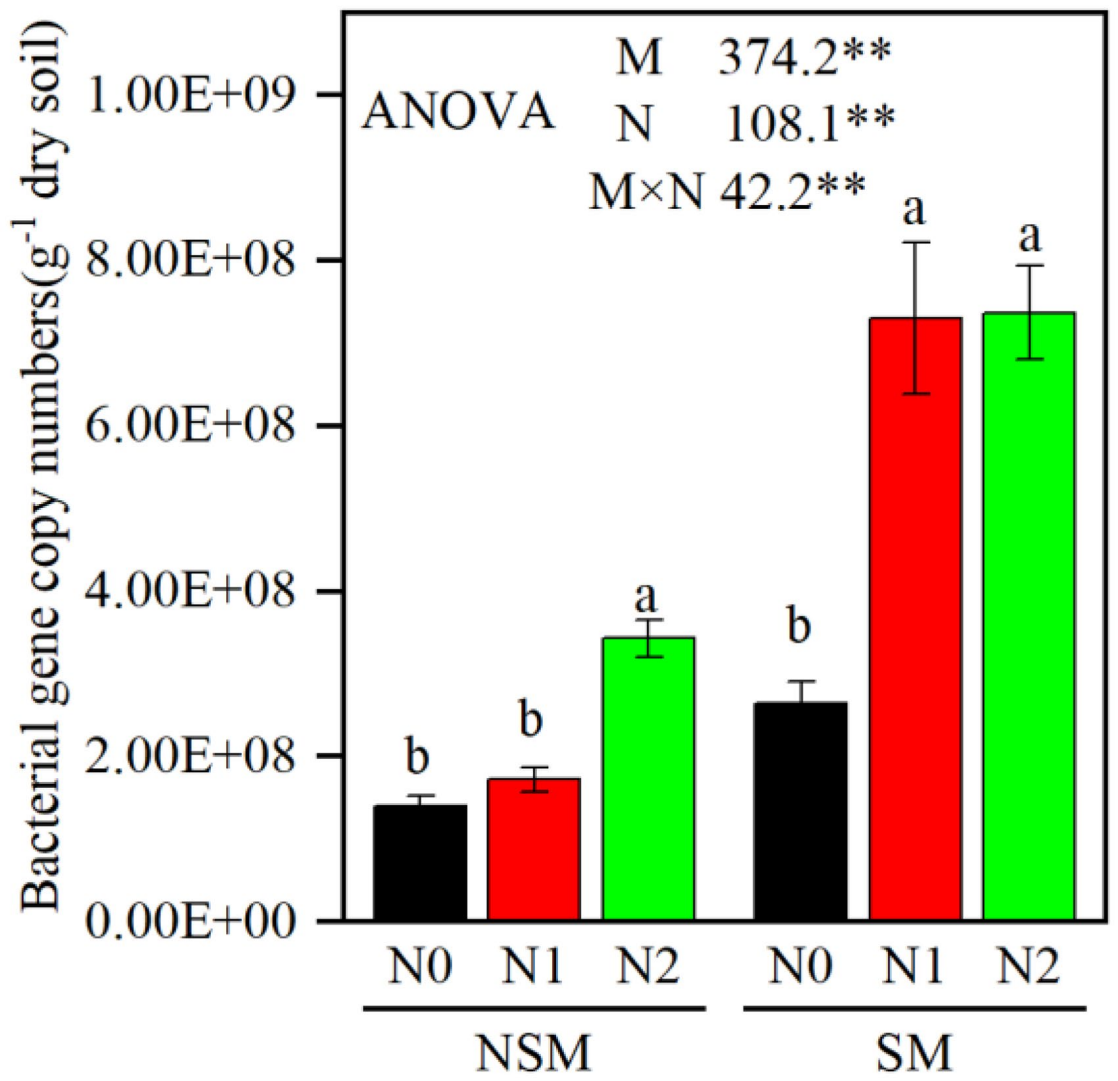
The abundance of bacteria gene copies in wheat rhizosphere soil under straw mulching and N fertilization treatments. M, mulching treatment; N, N fertilization treatment. M × N, the interaction of straw mulching and N fertilization NSM, no straw mulching; SM, straw mulching; N0, no N; N1, 120 kg N ha^−1^; N2, 180 kg N ha^−1^. Data shown as mean ± S.D. ** statistically significant difference (*p* < 0.01). Different letters above columns indicate statistically significant difference (*p* < 0.05) between N fertilizer levels within NSM and SM.

**Figure 2:**
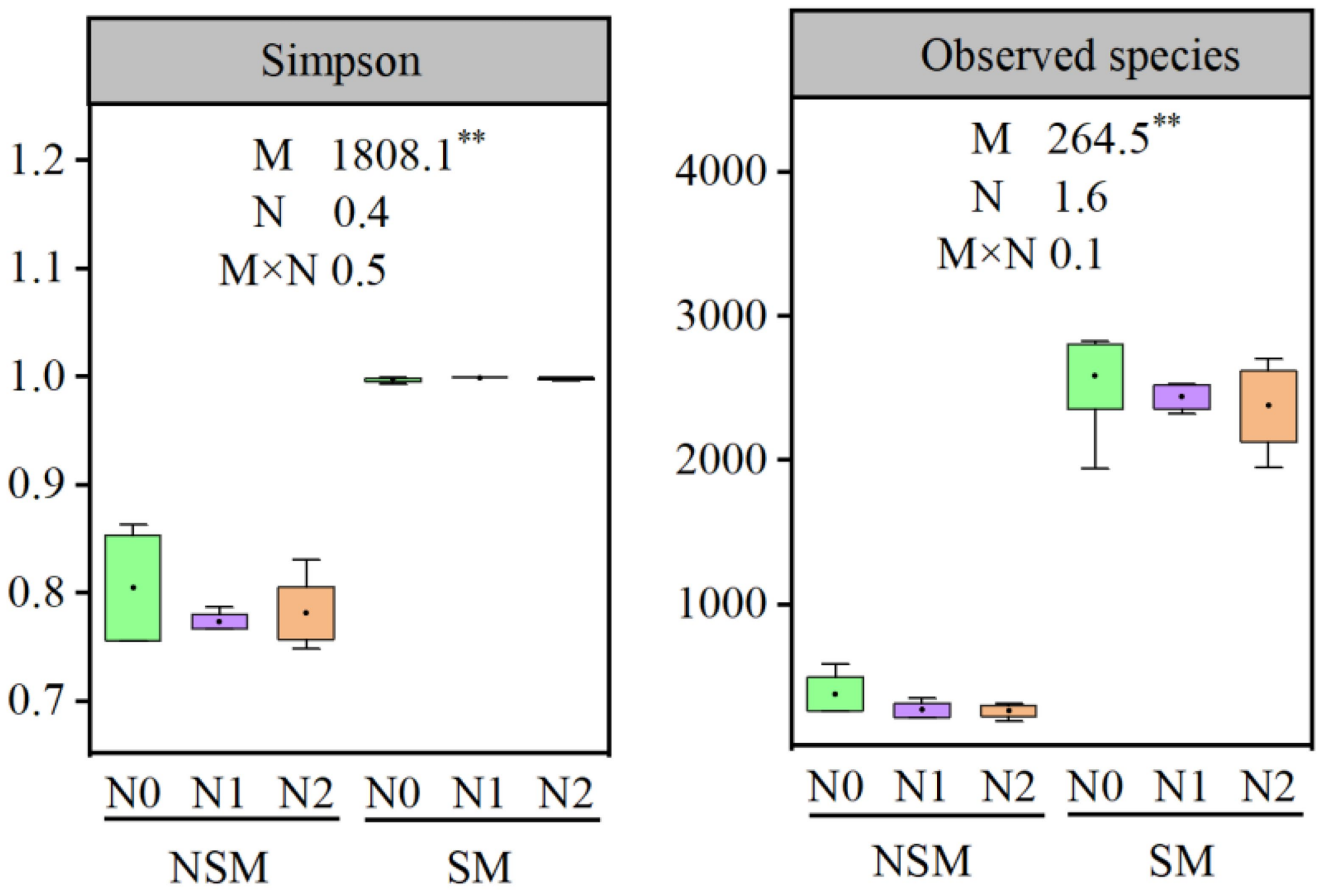
α-diversity of bacterial communities in wheat rhizosphere soil under straw mulching and N fertilization treatments. M, mulching treatment; N, N fertilization treatment. M × N, the interaction of straw mulching and N fertilization. NSM, no straw mulching; SM, straw mulching; N0, no N; N1, 120 kg N ha^−1^; N2, 180 kg N ha^−1^. **, statistically significant difference (*p* < 0.01).

**Figure 3:**
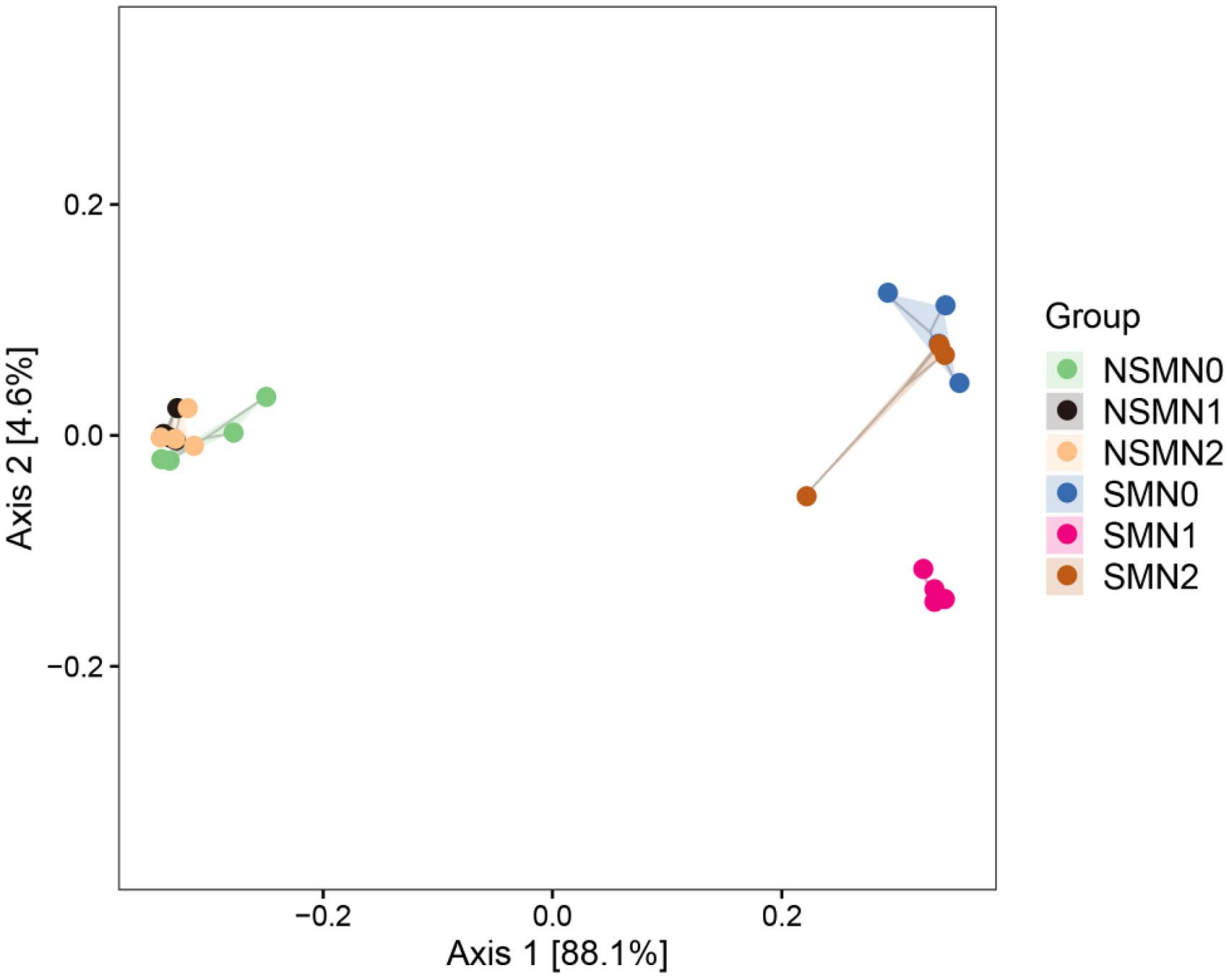
Principal coordinate analysis (PCoA) of microbial community composition in wheat rhizosphere soil under straw mulching and N fertilization treatments. NSM, no straw mulching; SM, straw mulching; N0, no N; N1, 120 kg N ha^−1^; N2, 180 kg N ha^−1^.

The 16S rRNA gene copy numbers ranged from 1.4 × 10^8^ to 7.4 × 10^8^ per g soil, with the lowest and the highest numbers in the NSMN0 and SMN2 treatments, respectively (Figure 1). The abundance and diversity of the rhizosphere bacterial communities were greater with than without mulching (*p* < 0.05; Figures 1, 2; Supplementary Tables S1–S4). The N fertilization level did not affect diversity. In line with the higher diversity with mulching, the number of identified genera was greater with mulching (67) than without (20) (Supplementary Table S4). In the PCoA, the rhizosphere bacterial communities from mulched and not mulched treatments were clearly separated along the PCoA axis1 that explained 88.1% of the variation in community composition (Figure 3). The communities in SMN1 were separated from those in SMN0 and SMN2 along PCoA axis2 that explained 4.6% of the variation. In line with the PCoA, the communities in the SMN0, SMN1 and SMN2 treatments were different from those without mulching and different from each other (Supplementary Table S6).

**Figure 4:**
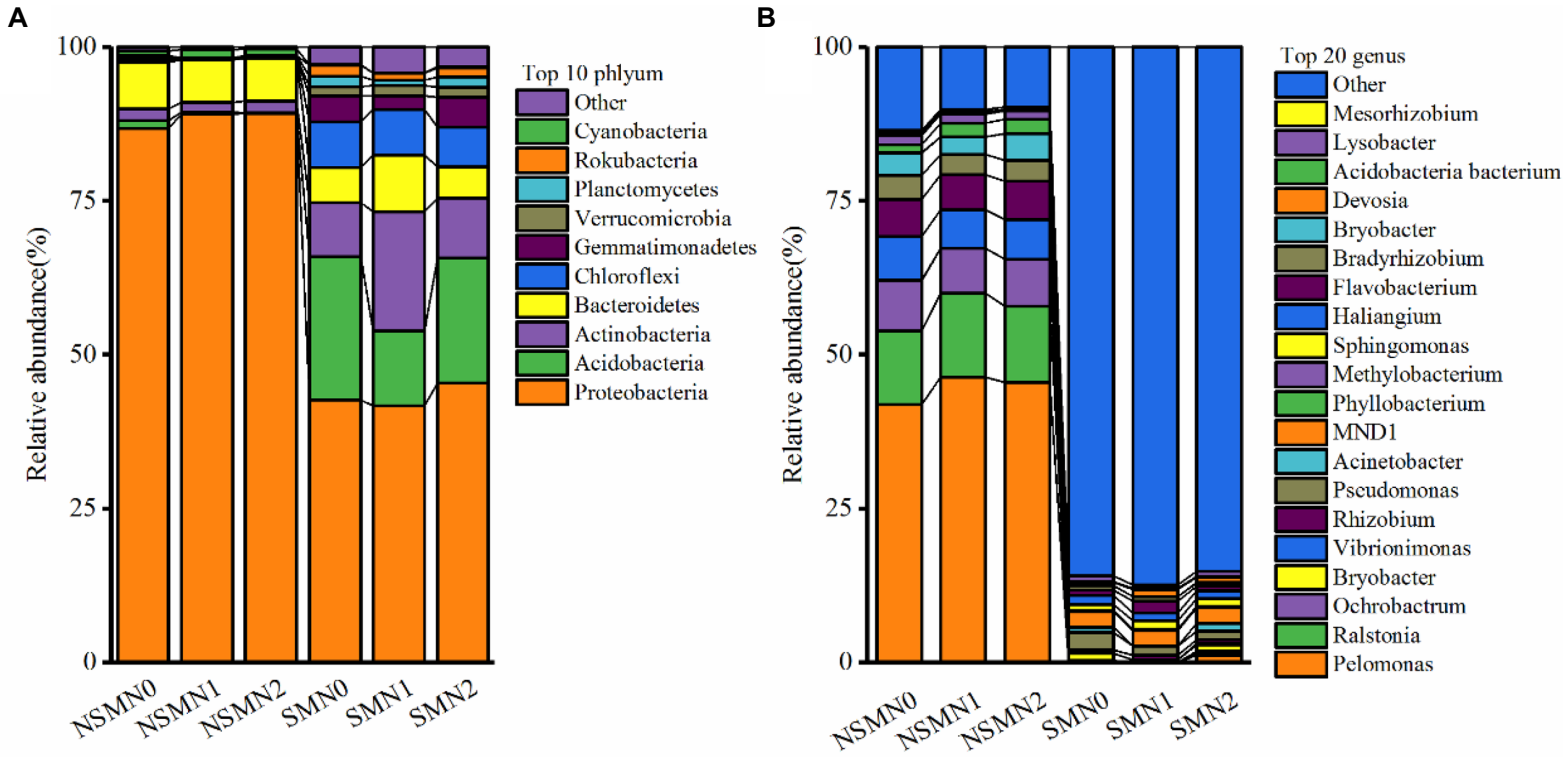
Bacterial community composition in wheat rhizosphere soil under straw mulching and N fertilization treatments. **(A)**, phylum level; **(B)**, genus level. NSM, no straw mulching; SM, straw mulching; N0, no N; N1, 120 kg N ha^−1^; N2, 180 kg N ha^−1^.

The relative abundances of phyla Proteobacteria and Bacteroidetes were high in all treatments and those of Acidobacteria, Actinobacteria and Chloroflexi in the mulched treatments (Figure 4A; Supplementary Table S3). The relative abundances of genera *Pelomonas*, *Ralstonia*, *Ochrobactrum* and *Vibrionimonas* were approximately 1% or less with mulching and ranged from over 40% to over 6% without mulching (Figure 4B; Supplementary Table S4).

### Characteristic taxa in the bacterial communities

**Figure 5:**
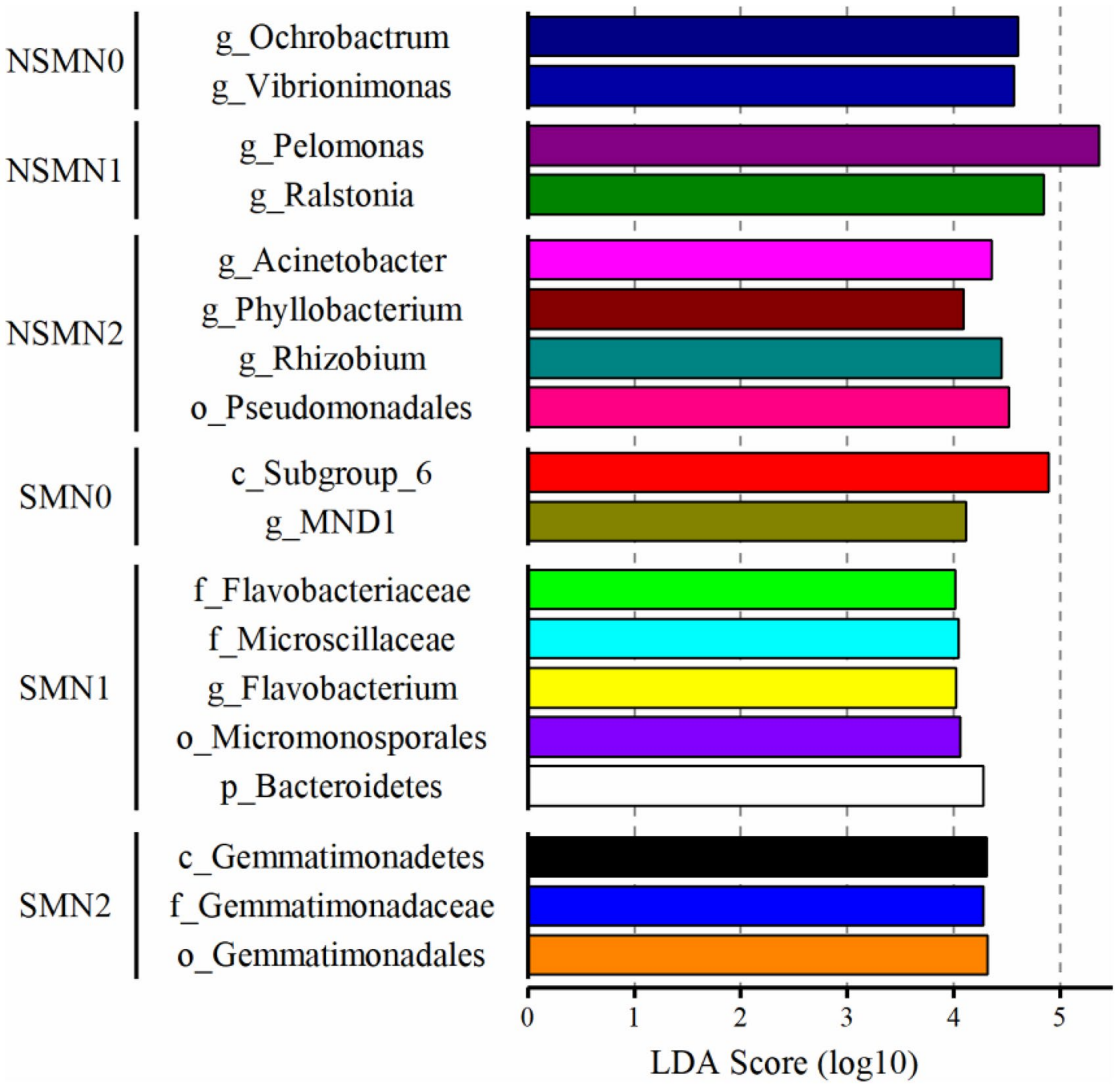
Differentially abundant taxa in wheat rhizosphere soil under straw mulching and N fertilization treatments. Detected using linear discriminant analysis effect size analysis. NSM, no straw mulching; SM, straw mulching; N0, no N; N1, 120 kg N ha^−1^; N2, 180 kg N ha^−1^.

The effect of straw mulching and N fertilization on individual taxa was assessed using LEfSe. In the differential abundance analysis, 154 and 35 OTUs had LDA scores >2.0 in the SM and NSM, respectively (Supplementary Table S5), and were considered as differentially abundant. Most of the differentially abundant OTUs in SM were assigned to Proteobacteria (51), Actinobacteria (22), Acidobacteria (16) and Chloroflexi (14), and in NSM to Proteobacteria (20) (Supplementary Table S5). The taxa characterizing the treatments with LDA score > 4 included class *Subgroup 6* belonging to Acidobacteria and proteobacterial order MND1 in the SMN0 treatment, phylum *Bacteroidetes* and actinobacterial order Micromonosporales in the SMN1 treatment, class *Gemmatimonadetes* in the SMN2 treatment, genera *Ochrobactrum* (Proteobacteria) and *Vibrionimonas* (Bacteroidetes) in the NSMN0 treatment, proteobacterial genera *Pelomonas* and *Ralstonia* in the NSMN1 treatment and proteobacterial order *Pseudomonadales* and genera *Acinetobacter*, *Phyllobacterium* and *Rhizobium* in the NSMN2 treatment (Figure 5).

### Relationships among bacterial parameters, soil properties, and wheat yield and biomass

**Figure 6:**
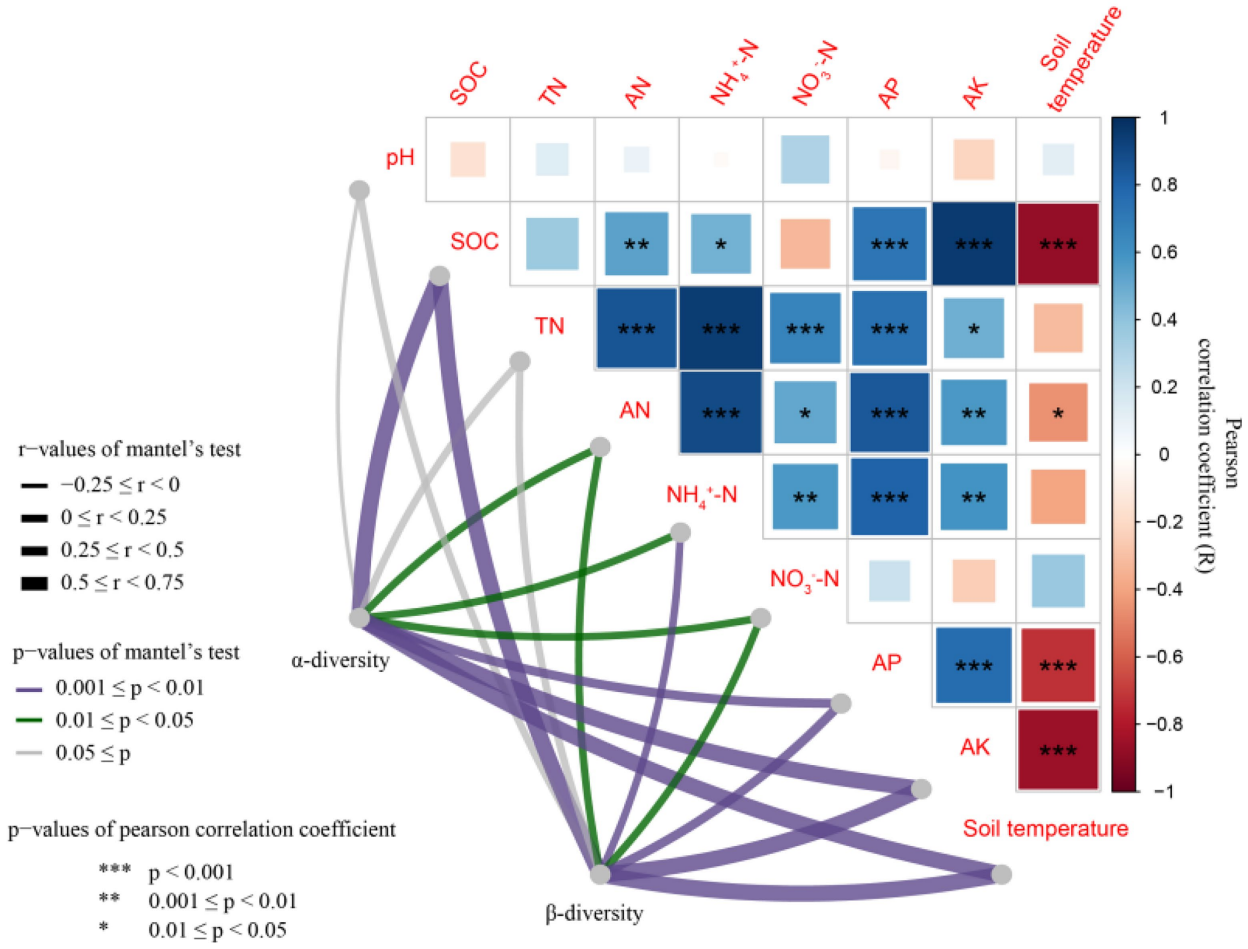
Relationship between bacterial α-diversity and β-diversity and soil environmental factors. SOC, Soil organic C; TN, Total N; AN, Available N; NH_4_^+^-N, Ammonium N; NO_3_^−^-N, Nitrate N; AP, Available phosphorus; AK, Available potassium.

**Figure 7:**
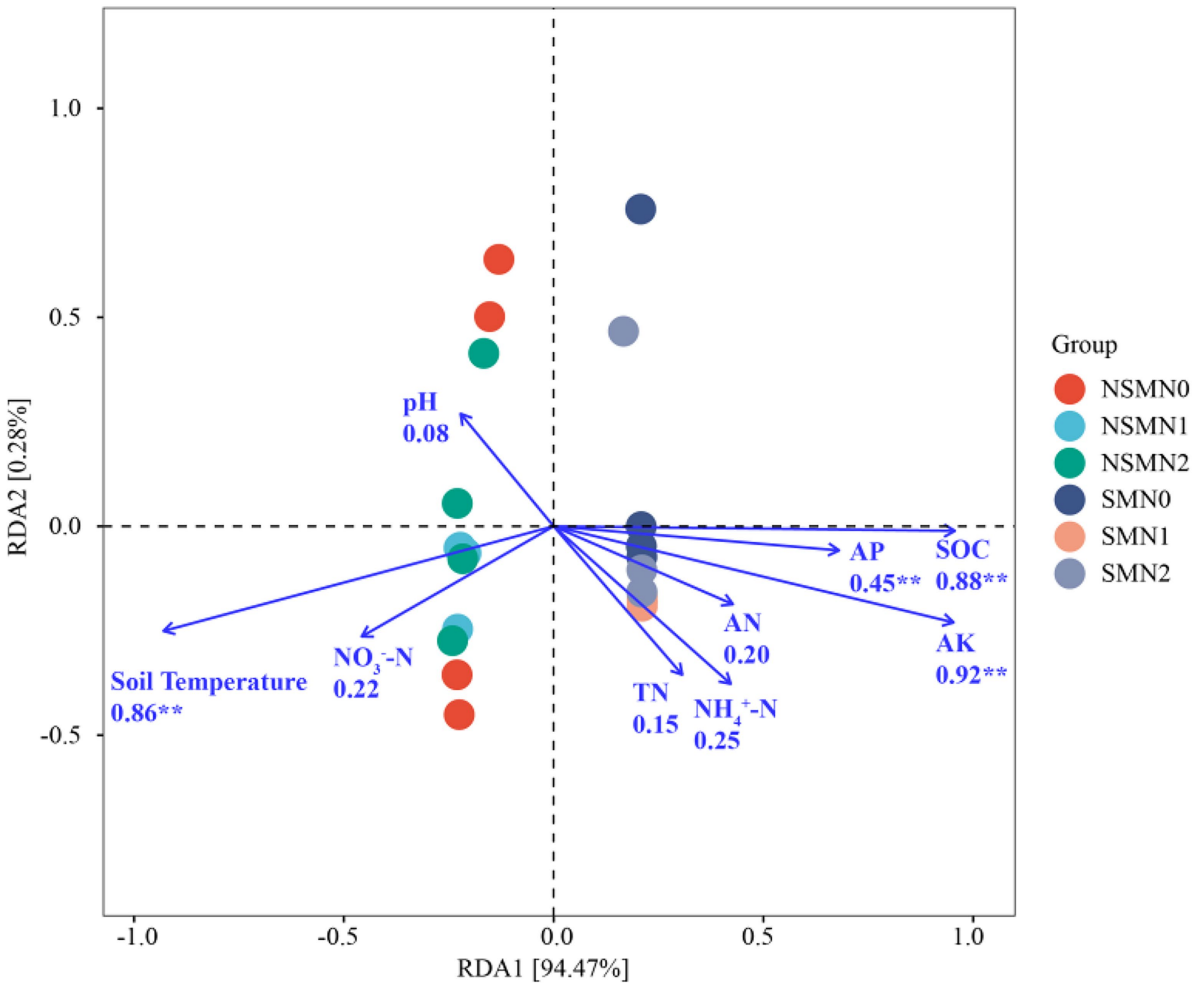
The relationships between environmental factors and bacterial communities in wheat rhizosphere soil under straw mulching and N fertilization treatments. NSM, no straw mulching; SM, straw mulching; N0, no N; N1, 120 kg N ha^−1^; N2, 180 kg N ha^−1^. SOC, Soil organic C; TN, Total N; AN, Available N; NH_4_^+^-N, Ammonium N; NO_3_^−^-N, Nitrate N; AP, Available phosphorus; AK, Available potassium. ^**^ indicates statistically significant difference at *p* < 0.01.

There was a significant correlation between most soil properties (*p* < 0.05), but none of the other properties correlated with soil pH (Figure 6). The differences in rhizosphere bacterial community composition were related to differences in soil temperature and SOC, AP and AK contents (*p* < 0.01; Figure 7). Soil temperature and SOC and AK contents correlated strongly (0.5 ≤ r < 0.75, *p* < 0.01), and AP, AN, NH_4_^+^-N and NO_3_^−^-N contents weakly (0 < r < 0.5, *p* < 0.05 or 0.01) with the α-diversity and β-diversity in the Mantel test (Figure 6). Similarly, Simpson diversity and the number of observed species correlated positively with wheat shoot biomass and yield (Table 2; Supplementary Figure S1). The abundance of 16S rRNA gene correlated positively with wheat shoot biomass and yield (*p* < 0.01).

**Table 2:** The correlations of bacteria α-diversity and gene abundance with biomass and wheat yield.

Index	Simpson	Observed species	Bacterial gene abundance
Shoot biomass	0.62^**^	0.63^**^	0.87^**^
Yield	0.51^**^	0.50^*^	0.91^**^

^**^ and ^*^ indicate statistically significant correlation at p < 0.01 and p < 0.05, respectively.

## Discussion

Straw mulching and N fertilization promote wheat yield, yet their combined effects on wheat rhizosphere bacterial communities remain largely unknown. Straw mulching is beneficial in maintaining soil health (Akhtar et al., 2019b). In our study, mulching affected both the composition and diversity of the bacterial communities. Straw mulching provides a C source for the soil microbes, allowing them to proliferate and diversify (Maarastawi et al., 2018). The additional nutrient input provided by the straw (Chen et al., 2017) may have led to the observed higher diversity and richness in the mulched treatment. In bulk soils, the α-diversities of bacteria were lower after long-term N fertilization (Zhou et al., 2015; Zeng et al., 2016). In a long-term N fertilization experiment, Wang et al. (2019) found that fertilization affected the α-diversity in the bulk soil but not in the rhizosphere. Similarly, in maize rhizosphere N fertilization affected root endophytic but not rhizospheric bacterial communities (Miranda-Carrazco et al., 2022). In agreement, in our study the diversities in the rhizosphere were similar across N fertilization levels. The difference in the response to N fertilization between bulk and rhizosphere soils may be due to the differences in their structural and physicochemical properties (Lorenz et al., 1994; Whalley et al., 2005). In line with our hypothesis, compared with sole N fertilization, straw mulching combined with N fertilization was more conducive to promote bacterial α-diversity and the increase of wheat yield in our study.

Several soil environmental factors, for example, pH, nutrient content and temperature, affect bacterial community composition and growth (Pietikainen et al., 2005; Li et al., 2014; Wang et al., 2017; Cheng et al., 2019). Zhao et al. (2019) suggested that C input and organic C content are critical for determining the bacterial community structure. The addition of straw increased SOC content and provided energy for microbial growth in low-C soil (Xiao et al., 2016). Similarly, in our study the SOC content was the main factor associated with differences in the wheat rhizosphere bacterial community composition. As the soil microbial communities are mainly C, not N limited (Soong et al., 2019), it is likely that the higher SOC content in the mulched treatment provided an effective C source for the bacteria. In previous studies the bacterial community composition correlated with the soil AP and AK that were possibly released into the soil due to the decomposition of the straw (Peng et al., 2016; Liang et al., 2020). In agreement, AP and AK contents were among the key factors related to differences in community composition in our study. In addition, the community composition correlated with soil temperature that was lower in the mulched treatment, possibly due to the effect of mulching on the soil albedo which is known to decrease the soil temperature (Kader et al., 2019).

As in Zhang et al. (2017), the Proteobacteria comprised the largest part of the relative abundance in all treatments. Most of the differentially abundant OTUs were proteobacterial as well. Overall, mulching resulted in a greater number of enriched OTUs than N fertilization. Multiple OTUs assigned to Acidobacteria, Chloroflexi and Actinobacteria, i.e., phyla considered key groups in the decomposition of organic matter in soil and of great significance in C turnover (Banerjee et al., 2016; Lewin et al., 2016), were enriched in the mulched treatment, further suggesting that the differences in rhizospheric communities were due to the availability of C. Among the most discriminative taxa in the mulched treatment, Acidobacteria Subgroup 6 includes putative plant growth promoters (PGP; Chen L. et al., 2021). All but one, the genus Vibrionimonas, of the most discriminative taxa in NSM were proteobacterial; the taxa included genus *Ralstonia*, a potential plant pathogen (Peeters et al., 2013), and taxa with possible plant growth promoting properties. The genera *Acinetobacter* and *Phyllobacterium* and order Pseudomonadales include P-solubilizing strains, and in addition to N fixation ability, IAA production is one of the characteristics of genus Rhizobium (Badenoch-Jones et al., 1982). Even though the not mulched soils were characterized by both PGP and pathogenic taxa, together with the higher abundance and diversity in the mulched treatment, the results implied that the mulching generally improved soil health.

## Conclusion

In line with our hypothesis, straw mulching for four years mulching increased the diversity and abundance of the bacterial communities in wheat rhizosphere. The straw mulching affected the composition of rhizospheric bacterial communities, likely mostly due to the increase in SOC content that provided an effective C source for the bacteria. The results offer valuable information for soil management strategies to shape the composition and function of soil bacterial communities in agroecosystems.

## Data availability statement

The datasets presented in this study can be found in online repositories. The names of the repository/repositories and accession number(s) can be found at: https://www.ncbi.nlm.nih.gov/, SRP297976.

## Author contributions

SC and GF contributed to conception and design of the study. XX organized the database. HM, TZ, and XH performed the statistical analysis. SC wrote the first draft of the manuscript. PP wrote sections of the manuscript. All authors contributed to manuscript revision, read, and approved the submitted version.

## Funding

This work was supported by Sichuan Science and Technology Program (2021YFYZ0002), Agro-scientific research in the public interest (20150312705), and Special Fund for National Key Research and Development Program (2016YFD0300406).

## Conflict of interest

The authors declare that the research was conducted in the absence of any commercial or financial relationships that could be construed as a potential conflict of interest.

## Publisher’s note

All claims expressed in this article are solely those of the authors and do not necessarily represent those of their affiliated organizations, or those of the publisher, the editors and the reviewers. Any product that may be evaluated in this article, or claim that may be made by its manufacturer, is not guaranteed or endorsed by the publisher.

## Footnotes

^1^ https://docs.qiime2.org/2019.4/tutorials/

^2^ http://huttenhower.sph.harvard.edu/lefse/

^3^ https://github.com/houyunhuang/ggcor

^4^ https://github.com/taiyun/corrplot
