# Supplementary material for: Response of soil bacterial communities in wheat rhizosphere to straw mulching and N fertilization
Source: Front Microbiol. 2022 Dec 9;13:982109. doi: 10.3389/fmicb.2022.982109 (PMC9780536; doi:10.3389/fmicb.2022.982109)
Supplement: Supplementary file 1 [file Data_Sheet_1.PDF]

Response of Soil Bacterial Communities in Wheat Rhizosphere to Straw Mulching and N Fertilization

Songhe Chen[1,2], Xiaoling Xiang[1], Hongliang Ma[1], Petri Penttinen[3], Ting Zheng[1], Xiulan Huang[1], Gaoqiong Fan[1*]

[1] Key Laboratory of Crop Eco-Physiology & farming system in Southwest China, Ministry of Agriculture, College of Agronomy, Sichuan Agricultural University, Chengdu, 611130, Sichuan Province, PR China; [2] Root Biology Center, College of Resources and Environment, Fujian Agriculture and Forestry University, Fuzhou, 350002 China; [3] Department of Microbiology College of Resources, Sichuan Agricultural University, Chengdu, 611130, Sichuan Province, PR China

[*]Corresponding author: fangao20056@126.com (G. Fan.)

SUPPLEMENTARY INFORMATION LIST

SUPPLEMENTARY TABLES

Supplementary Table S1
Supplementary Table S2
Supplementary Table S3
Supplementary Table S4
Supplementary Table S5
Supplementary Table S6

SUPPLEMENTARY FIGURES

Supplementary Fig.S1

SUPPLEMENTARY MATERIAL

Supplementary Table S1 Diversity indexes of 24 soil samples.

Sample	Simpson	Observed species
NSMN01	0.756306	257.9
NSMN02	0.755631	267.4
NSMN03	0.862989	402.1
NSMN04	0.843695	588.6
NSMN11	0.786843	352.3
NSMN12	0.767498	226.4
NSMN13	0.766693	273.9
NSMN14	0.773603	213.5
NSMN21	0.780271	310.7
NSMN22	0.831014	288.5
NSMN23	0.747839	266
NSMN24	0.76616	186.2
SMN01	0.999012	2791.9
SMN02	0.99891	2828.1
SMN03	0.998951	2778.6
SMN04	0.993258	1939.4
SMN11	0.998882	2321.7
SMN12	0.999002	2526.1
SMN13	0.998997	2527.4
SMN14	0.998924	2396.8
SMN21	0.998976	2702.1
SMN22	0.998825	2314.1
SMN23	0.998902	2542.3
SMN24	0.996183	1950

Supplementary Table S2 Analysis of variance of diversity indexes

Straw mulching	Nitrogen level	Simpson	Observed species
NSM	N0	0.805b	379b
	N1	0.774b	267b
	N2	0.781b	263b
SM	N0	0.998a	2585a
	N1	0.999a	2443a
	N2	0.998a	2377a
F-value	M	1808.3**	264.5**
	N	0.4	1.6
	M×N	0.5	0.1

NSM, no straw mulching; SM, straw mulching; N0, no nitrogen; N1, 120 kg N ha^{-1}; N2, 180 kg N ha^{-1}; M, mulching treatment; N, nitrogen fertilization treatment. For each variable, different letters indicate significant difference between treatments at $P<0.05$. **, statistically significant difference ($P < 0.01$).

Supplementary Table S3 The relative abundances of major phyla in wheat rhizosphere soil in the straw mulching and N fertilization treatments

phylum	NSMN0	NSMN1	NSMN2	SMN0	SMN1	SMN2
Proteobacteria	86.710	89.116	89.158	42.577	41.651	45.375
Acidobacteria	1.304	0.268	0.156	23.357	12.204	20.288
Actinobacteria	1.855	1.564	1.843	8.793	19.332	9.774
Bacteroidetes	7.644	6.870	6.979	5.684	9.195	5.052
Chloroflexi	0.429	0.066	0.125	7.385	7.464	6.479
Gemmatimonadetes	0.293	0.057	0.012	4.205	2.241	4.818
Verrucomicrobia	0.041	0.025	0.025	1.507	1.689	1.670
Planctomycetes	0.171	0.209	0.260	1.650	0.843	1.681
Rokubacteria	0.101	0.008	0.002	1.831	1.079	1.500
Cyanobacteria	0.828	1.385	1.147	0.139	0.068	0.142
Patescibacteria	0.044	0.010	0.021	0.505	1.885	0.690
Latescibacteria	0.040	0.000	0.000	0.625	0.626	0.727
Firmicutes	0.324	0.245	0.132	0.413	0.429	0.418
Nitrospirae	0.038	0.012	0.010	0.612	0.414	0.490
Entotheonellaeota	0.013	0.000	0.005	0.244	0.117	0.172
Fibrobacteres	0.000	0.000	0.000	0.038	0.231	0.240
Armatimonadetes	0.039	0.023	0.041	0.110	0.095	0.082
Chlamydiae	0.008	0.008	0.000	0.050	0.163	0.104
Dependentiae	0.011	0.000	0.000	0.042	0.075	0.066
Deinococcus-Thermus	0.048	0.071	0.049	0.004	0.003	0.002
Elusimicrobia	0.003	0.000	0.005	0.011	0.043	0.023
WS2	0.000	0.000	0.000	0.025	0.008	0.024
WPS-2	0.028	0.044	0.020	0.000	0.003	0.008
Fusobacteria	0.014	0.018	0.004	0.000	0.000	0.000
Spirochaetes	0.003	0.000	0.000	0.023	0.003	0.039
BRC1	0.000	0.000	0.000	0.035	0.019	0.040
Other	0.010	0.004	0.007	0.135	0.120	0.097

Supplementary Table S4 The relative abundances of major genera in wheat rhizosphere soil in the straw mulching and N fertilization treatments

genus	NSMN0	NSMN1	NSMN2	SMN0	SMN1	SMN2
Pelomonas	41.918	46.369	45.464	0.078	0.000	1.151
Ralstonia	11.879	13.623	12.384	0.098	0.000	0.396
Ochrobactrum	8.195	7.244	7.583	0.137	0.000	0.265
Bryobacter	0.106	0.080	0.071	1.102	0.355	0.983
Vibrionimonas	7.129	6.169	6.475	0.086	0.000	0.229
Allorhizobium-Neorhizobium-Pararhizobium-Rhizobium	6.010	5.796	6.198	0.511	0.817	0.603
Pseudomonas	3.853	3.250	3.347	2.802	1.437	1.419
Acinetobacter	3.627	2.847	4.381	0.922	0.009	1.255
MND1	0.065	0.017	0.001	2.526	2.502	2.543
Phyllobacterium	1.336	2.249	2.338	0.054	0.004	0.097
Methylobacterium	1.433	1.465	1.310	0.120	0.193	0.158
Sphingomonas	0.402	0.329	0.245	0.944	1.410	1.194
Haliangium	0.064	0.022	0.005	1.482	1.280	1.360
Flavobacterium	0.023	0.040	0.012	0.846	1.909	0.679
Bradyrhizobium	0.346	0.340	0.389	0.775	0.767	0.600
Bryobacter	0.001	0.001	0.001	0.011	0.004	0.010
Devosia	0.043	0.003	0.042	0.494	1.172	0.894
Acidobacteria bacterium	0.000	0.000	0.000	0.086	0.126	0.138
Lysobacter	0.025	0.008	0.005	0.932	0.582	0.808
Mesorhizobium	0.004	0.004	0.004	0.003	0.004	0.003
Micromonospora	0.000	0.000	0.000	0.141	0.551	0.287
Luteitalea	0.000	0.000	0.000	0.318	0.150	0.332
Demequina	0.000	0.000	0.000	0.250	0.340	0.284
Parafrigoribacterium	0.000	0.000	0.000	0.059	0.213	0.114
Virgisporangium	0.000	0.000	0.000	0.138	0.254	0.025
Actinocorallia	0.000	0.000	0.000	0.052	0.376	0.426
Flavihumibacter	0.000	0.000	0.000	0.126	0.118	0.082
Terrimonas	0.000	0.000	0.000	0.158	0.390	0.147
Adhaeribacter	0.000	0.000	0.000	0.121	0.147	0.087
Chryseolinea	0.000	0.000	0.000	0.295	0.478	0.302
OLB12	0.000	0.000	0.000	0.082	0.095	0.042
Ohtaekwangia	0.000	0.000	0.000	0.234	0.473	0.173
Dyadobacter	0.000	0.000	0.000	0.143	0.138	0.092
Mucilaginibacter	0.000	0.000	0.000	0.025	0.186	0.065
Solitalea	0.000	0.000	0.000	0.157	0.113	0.064
OLB13	0.000	0.000	0.000	0.059	0.037	0.097
Candidatus Chloroploca	0.000	0.000	0.000	0.031	0.149	0.020
AKYG587	0.000	0.000	0.000	0.213	0.137	0.297
Hirschia	0.000	0.000	0.000	0.109	0.192	0.169
Microvirga	0.000	0.000	0.000	0.076	0.119	0.112

Kaistia	0.000	0.000	0.000	0.060	0.070	0.059
Ellin6055	0.000	0.000	0.000	0.122	0.140	0.139
Ensifer	0.000	0.000	0.000	0.519	0.164	0.139
Bauldia	0.000	0.000	0.000	0.038	0.138	0.075
Pseudorhodoplanes	0.000	0.000	0.000	0.068	0.217	0.105
Sphingopyxis	0.000	0.000	0.000	0.030	0.107	0.074
Archangium	0.000	0.000	0.000	0.062	0.066	0.011
Starkeya	0.000	0.000	0.000	0.066	0.053	0.045
Lautropia	0.000	0.000	0.000	0.071	0.054	0.073
Sorangiineae bacterium	0.000	0.000	0.000	0.044	0.047	0.070
Methylibium	0.000	0.000	0.000	0.074	0.049	0.058
Piscinibacter	0.000	0.000	0.000	0.180	0.329	0.207
Pseudorhodoferax	0.000	0.000	0.000	0.020	0.150	0.061
Variovorax	0.000	0.000	0.000	0.027	0.133	0.025
Xylophilus	0.000	0.000	0.000	0.114	0.123	0.111
IS-44	0.000	0.000	0.000	0.040	0.126	0.068
Nitrosospira	0.000	0.000	0.000	0.085	0.135	0.126
mle1-7	0.000	0.000	0.000	0.264	0.193	0.254
Azospira	0.000	0.000	0.000	0.122	0.096	0.074
Azovibrio	0.000	0.000	0.000	0.033	0.067	0.169
Cellvibrio	0.000	0.000	0.000	0.109	0.329	0.289
OM60(NOR5) clade	0.000	0.000	0.007	0.097	0.045	0.104
Aquicella	0.000	0.000	0.000	0.136	0.035	0.090
BIyi10	0.000	0.000	0.000	0.031	0.134	0.110
Polycyclovorans	0.000	0.000	0.000	0.197	0.147	0.256
Candidatus Xiphinematobacter	0.000	0.000	0.000	0.071	0.059	0.050
Roseimicrobium	0.000	0.000	0.000	0.118	0.156	0.169
Serratia	0.083	0.057	0.080	0.000	0.000	0.000
others	13.460	10.088	9.658	80.406	79.412	78.981

Supplementary Table S5 Number of OTUs with LDA>2 in the phyla

treatment	Phylum	N. Clade
SMN0	Proteobacteria	26
	Acidobacteria	13
	Chloroflexi	6
	Nitrospirae	5
	Entotheonellaeota	4
	Planctomycetes	3
	Firmicutes	3
	Rokubacteria	3
	Verrucomicrobia	3
	Bacteroidetes	1
	Armatimonadetes	1
SMN1	Actinobacteria	22
	Proteobacteria	16
	Bacteroidetes	9
	Chloroflexi	8
	Patescibacteria	4
	Chlamydiae	3
	Verrucomicrobia	2
	Elusimicrobia	1
SMN2	Proteobacteria	9
	Acidobacteria	3
	Verrucomicrobia	3
	Gemmatimonadetes	3
	Planctomycetes	2
	Cyanobacteria	1
NSMN0	Proteobacteria	4
	Deinococcus_Thermus	3
	Phreatobacter	1
	Bacteroidetes	1
NSMN1	Proteobacteria	6
	Cyanobacteria	3
	Bacteroidetes	2
	Deinococcus_Thermus	2
	Actinobacteria	1
NSMN2	Proteobacteria	10
	Planctomycetes	1
	Cyanobacteria	1

Supplementary Table S6 Analysis of significant difference between groups

Group 1	Group 2	Permutations	q-value
NSMN0	NSMN1	999	0.53
NSMN0	NSMN2	999	0.64
NSMN0	SMN0	999	0.04
NSMN0	SMN1	999	0.04
NSMN0	SMN2	999	0.04
NSMN1	NSMN2	999	0.79
NSMN1	SMN0	999	0.04
NSMN1	SMN1	999	0.04
NSMN1	SMN2	999	0.04
NSMN2	SMN0	999	0.04
NSMN2	SMN1	999	0.04
NSMN2	SMN2	999	0.04
SMN0	SMN1	999	0.04
SMN0	SMN2	999	0.04
SMN1	SMN2	999	0.04

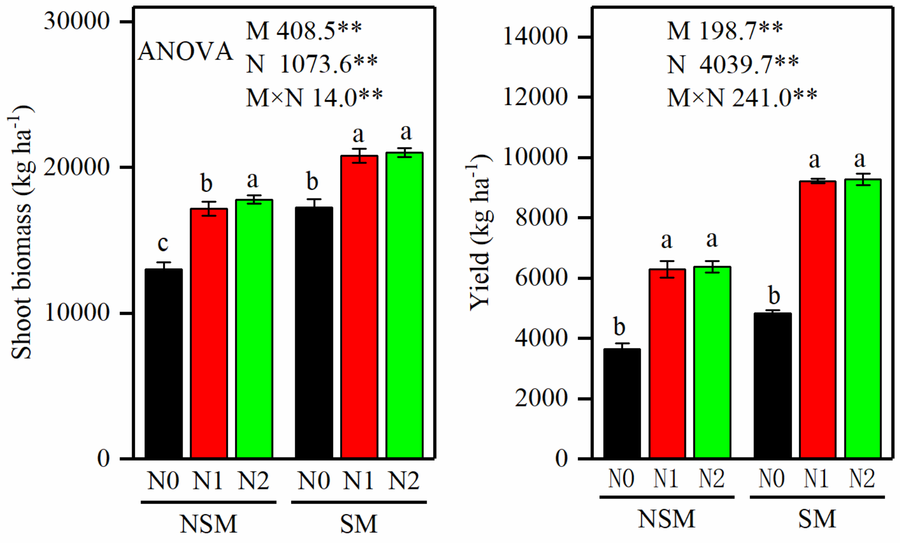

Supplementary Fig.S1 Shoot biomass and yield of wheat under straw mulching and nitrogen fertilization. NSM, no straw mulching; SM, straw mulching; N0, no nitrogen; N1, 120 kg N ha^{-1}; N2, 180 kg N ha^{-1}. Data shown as mean ± S.D. ** indicates statistically significant difference ($P < 0.05$) between NSM and SM. Different letters above columns indicate statistically significant difference ($P < 0.05$) between N fertilizer levels within NSM and SM.
